# Versatile and Scalable Nanoparticle Vaccine as a Scaffold Against Newly Emerging Influenza Viruses

**DOI:** 10.3390/v17091165

**Published:** 2025-08-26

**Authors:** Alessandro Pardini, Dominik A. Rothen, Pascal S. Krenger, Anne-Cathrine Vogt, Romano Josi, Xuelan Liu, Kaspars Tars, Manfred Kopf, Monique Vogel, Martin F. Bachmann

**Affiliations:** 1 Department of BioMedical Research, University of Bern, 3008 Bern, Switzerland; 2 Department of Rheumatology and Immunology, University Hospital Bern, 3008 Bern, Switzerland; 3 Graduate School of Cellular and Biomedical Sciences, University of Bern, 3008 Bern, Switzerland; 4 International Immunology Centre, Anhui Agricultural University, Hefei 230036, China; 5 Latvian Biomedical Research & Study Centre, Ratsupites iela 1, LV 1067 Riga, Latvia; 6 Institute of Molecular Health Sciences, ETH Zurich, 8093 Zurich, Switzerland; 7 Nuffield Department of Medicine, Centre for Cellular and Molecular Physiology (CCMP), The Jenner Institute, University of Oxford, Oxford OX3 7DQ, UK

**Keywords:** vaccine, influenza, virus-like particle, public health

## Abstract

Influenza remains a major health threat due to its high contagiousness and global spread, affecting not only humans but also agricultural livestock and wild animals through transmission via migratory birds. Despite over 70 years of vaccination, influenza still creates epidemics and pandemics, and the ongoing use of vaccination is an essential but currently insufficient strategy. In this study, we assessed the immunogenicity and efficacy of an AP205 virus-like particle (VLP) carrying the HA head domain of the A/PR8/H1N1 strain, administered intranasally and subcutaneously in mice. For this purpose, the entire head region of A/PR8/H1N1 was genetically integrated into a sterically improved version of AP205, which exhibits capsid monomers fused into a dimer, thereby offering inexpensive and scalable production processes. The vaccine induced strong systemic anti-HA IgG and IgA antibodies via both routes, with no significant difference in the levels of IgG. Both immunisation strategies induced protection against a lethal challenge with H1PR8 mouse-adapted influenza virus. The findings demonstrate the potential of the AP205 VLP platform for HA1-based influenza vaccines and its applicability for controlling influenza in both humans and livestock.

## 1. Introduction

Influenza is an infectious respiratory illness caused in humans by the influenza A and influenza B viruses. Most individuals affected by influenza exhibit an abrupt onset of respiratory symptoms and myalgia, sometimes accompanied by fever, and typically recover within a week. However, a significant proportion of cases progress to severe and fatal pneumonia caused by either the virus itself or secondary bacterial infections [1,2]. Seasonal influenza, a recurring global phenomenon, affects one in five unvaccinated children and one in ten unvaccinated adults, whether or not they display symptoms [3]. Influenza circulates throughout the year, with sporadic outbreaks. Annually, influenza causes 1 billion cases, including 3–5 million severe ones and 290,000 to 650,000 respiratory-related deaths [4]. Higher-risk groups include individuals aged 65 and over, those afflicted with chronic illnesses, pregnant women, and children under the age of five [5]. Although influenza occurs in seasonal epidemics, occasional and unpredictable worldwide pandemic outbreaks can occur due to antigenic shifts, typically caused by the influenza A virus of zoonotic origin, or upon reassembly with a zoonotic influenza virus [1]. Prevention relies on annual influenza vaccination, with ongoing efforts to develop new, more effective, and better-targeted vaccines. Nonetheless, sporadic zoonotic infections from novel avian or swine influenza A viruses remain a persistent pandemic threat [2,6].

The complexity, unpredictability, and multi-species involvement of influenza epidemics necessitate a One Health approach to address the issue promptly and holistically. Key priorities include enhanced surveillance in animal populations and farming environments, the real-time genomic surveillance of H5 Highly Pathogenic Avian Influenza (HPAI) viruses, improvements in farm biosecurity, the vaccination of animals, and wildlife conservation practices that do not compromise habitat integrity [7,8].

Influenza viruses belong to the Orthomyxoviridae family and are classified into four distinct types: A, B, C, and D. Only influenza A and B viruses infect humans. They are characterised by eight negative-sense, single-stranded RNA gene segments, encoding ten essential viral proteins and additional strain-specific proteins [9]. Haemagglutinin (HA) is the primary viral surface antigen and the main target of neutralising antibodies. It is a trimer with two distinct extracellular domains called the head domain (HA1) and the stalk domain (HA2). HA1 is responsible for the receptor-binding site and variable antigenic determinants [10,11]. HA is essential for viral attachment and entry into the host cell via endocytosis and membrane fusion, making it a valuable target for both vaccines and therapies [12,13]. Influenza viruses evolve through antigenic drift and antigenic shift, making it difficult for a vaccine to be effective in the long term [14]. Current influenza vaccines are produced using egg-based, cell-based, or recombinant technologies. Inactivated vaccines represent the most widely used of these and come in various formulations, including whole virus, split-virion, subunit, virosome-based, live attenuated, and recombinant viral vector vaccines [4,12,15].

Virus-like particles (VLPs) are self-assembling systems, generating virus-shaped particles, produced by the expression of one or more viral proteins [16]. VLP-based vaccines are already commercially available against HPV, HBV, malaria, and most recently chikungunya. VLP-based vaccines have been extensively explored for use against different diseases such as COVID-19, influenza, malaria, allergy, neurodegenerative disease, cancer, and even for plant applications [17,18,19,20,21,22,23,24,25]. Because of the absence of a viral genome or replicases, VLPs are unable to replicate. Nonetheless, it has been observed that some VLPs can encapsulate host-derived RNA or DNA fragments [26]. The high immunogenicity of these particles is due to virus-like pathogen-associated structural patterns (PASPs), which include repetitive structures and particle size and sometimes pathogen-associated molecular patterns (PAMPs) represented by encapsulated RNA fragments. Noncoding RNA or DNA fragments co-packaged during the formation of VLPs in systems such as RNA bacteriophages have been demonstrated to interact with various pattern recognition receptors (PRRs), in particular TLRs, thereby enhancing the immunogenicity of these particles [16,26,27].

Here, we used the AP205 nanoparticle, based on the coat protein of the bacteriophage AP205 that infects Acinetobacter bacteria. Following the expression of the coat protein in *Escherichia coli*, AP205-VLPs form stable particles and have a robust coat protein that can accommodate large insertions in the C or N termini. The dimer structure is distinguished by the proximity of the C-terminus of one monomer to the N-terminus of the adjacent monomer. In comparison to other members of the same family, AP205 is unique in lacking a beta-strand at the N terminus and the presence of an additional beta strand at the C terminus [28]. Consequently, the N and C termini of AP205 are in the same position and are highly exposed to the surface.

To achieve the steric optimisation of the VLP, monomers were dimerized, producing a particle with 90 instead of 180 N- and C-termini. This reduction in termini provides more space for the fusion and proper folding of larger epitopes on the VLP surface, thereby enhancing the induction of strong antibody responses [20,23,28]. This structural feature also accounts for the VLP’s ability to tolerate and incorporate extended insertions at both termini without compromising capsid integrity [28,29].

Here, we have shown that an AP205dim fusion with the HA1 domain based on the H1N1 PR8 (A/Puerto Rico/8/1934(H1N1)) strain can be produced in *E. coli*. The vaccines were then tested using different immunisation routes in a murine model. The immunisation of mice resulted in the induction of specific antibodies as well as the ability of the induced antibodies to neutralise the virus in vitro. Furthermore, a challenge experiment with live virus was performed to investigate the different immunisation routes further, showing that vaccinated mice were protected against lethal infection with the H1N1 PR8 virus.

## 2. Materials and Methods

### 2.1. Mice Animals

All in vivo tests were conducted using 8- to 12-week-old wild-type (wt) female BALB/cOlaHsd mice (Envigo, Amsterdam, The Netherlands). All animal procedures were conducted in accordance with the Swiss Animal Welfare Act (455.109.1-5, September 2008) and approved by the Swiss Federal Veterinary Office. Experiments were performed at the University of Bern under the relevant institutional guidelines.

### 2.2. Development of HA1–AP205dim Vaccine

The amino acid sequence of influenza A virus (A/Puerto Rico/8/1934(H1N1)) HA1 head domain was obtained from GenBank (ID: 956529), N-terminally fused via a GSGG linker to an AP205dimer construct, and inserted into pET-28a(+) (Gene Universal Inc., Newark, NJ, USA) for bacterial expression. The plasmid was reconstituted to a final concentration of 50 µg/µL and transformed into electrocompetent XL-1 cells. After transformation, a single colony was picked and grown overnight at 37 °C, 200 rpm. For protein expression, bacteria were grown in LB medium until reaching an OD_600nm_ of 0.4–0.6, then induced with 1 mM IPTG for 4–6 h at 37 °C, 200 rpm. Cells were harvested via centrifugation at 5000× *g* for 25 min at 4 °C. The supernatant was discarded, and the pellet was resuspended at a ratio of 1 g pellet in 10 mL of lysis buffer (20 mM of tris, 5 mM of EDTA, and 5% glycerol). Cells were subsequently lysed via sonication and washed four times with lysis buffer containing lysozyme (Sigma Aldrich, Burlington, MA, USA, L2879-5G). Inclusion bodies were solubilised via incubation in solubilization buffer (8 M of urea, 50 mM of Tris-HCl, and 150 mM of NaCl) for 16 h at 4 °C on a rotating wheel. The solubilised protein was dialysed against refolding buffer (4 M of urea, 100 mM of Na_2_HPO_4_ 2 H_2_O, 100 mM of NAH_2_PO_4_, 0.5 M of arginine, 5 mM of reduced glutathione, and 0.5 mM of oxidised glutathione), gradually decreasing the urea concentration (2 M and then 0 M) over 96 h. The final dialysis step was performed in PBS for 24–48 h. The refolded protein solution was concentrated, sterile-filtered, and purified using Sephacryl S500 H26/60 size-exclusion chromatography (Cytiva, Marlborough, MA, USA). Fractions containing the protein of interest were analysed via SDS-PAGE (12%, stained with ReadyBlue, Protein Gel Stain (Sigma Aldrich, Burlington, MA, USA, RSB-1L), Western blot, and electron microscopy. SDS-PAGE gels were imaged using an Azure Biosystem c300 scanner (Azure Biosystems, 6747 Sierra Court, Dublin, CA, USA) using the visible channel.

### 2.3. SDS-PAGE Analysis

For protein analysis, 15 μL of sample was mixed with 3 μL of 5× reduction buffer (Thermo Scientific, Waltham, MA, USA, Cat. 39000). The sample was heated at 95 °C for 5 min. Samples were then loaded onto an SDS-PAGE (12% with 4% stacking gel). A protein ladder (10 μL, Thermo Scientific, Cat. 26616) was included for molecular weight reference. Electrophoresis was run in a buffer containing 25 mM of tris, 25 mM of glycine, and 25 mM of 0.1% SDS. Gels were run first at 70 V, then adjusted to the sample migration. Gels were then stained with InstantBlue^®^ Coomassie Protein Stain (Abcam, Cat. Ab119211, Cambridge, UK) and imaged using the Azure Biosystems c300 (Azure Biosystems, 6747 Sierra Court, Dublin, CA, USA).

### 2.4. Western Blot

For immunoblotting, 5 µg of each protein (AP205, HA1–AP205dim) was put onto a 12% SDS gel and then transferred to a 0.2 µm nitrocellulose membrane using the Bio-Rad Trans-Blot^®^ Turbo© Transfer System (Hercules, CA, USA). Western blotting was performed using the iBindTM Flex Western System (Number: SLF2000, Invitrogen, Waltham, MA, USA, Publication No. MAN0010926) according to the manufacturer’s instructions. A self-made α-AP205 IgG was used as the primary antibody at a 1:1000 dilution, and a horseradish peroxidase (HRP) anti-mouse IgG Fc gamma (Jackson Immunoresearch Cat. No. C840T69, West Grove, PA, USA, 1:1000) was used for detection.

### 2.5. Electron Microscopy

The integrity and physical appearance of the vaccine candidate HA1–AP205dim VLP was visualised via transmission electron microscopy (Philips CM12 EM, Philips Electronics, Amsterdam, The Netherlands). Sample grids were glow-discharged before incubating with 10 μL of HA1–AP205dim (1.1 mg/mL) for 30 s. The grids were then rinsed three times with ddH20 and negatively stained for 30 s with 5 μL of 5% uranyl acetate. Excess uranyl acetate was removed, and the sample grids were air-dried for 10 min. Images were captured at magnifications of 84,000× and 110,000×.

### 2.6. Immunisation and Virus Challenge

Wild-type BALB/cOlaHsd mice (8–12 weeks old) were randomly assigned to receive either the HA1–AP205dim vaccine or the AP205dim control and were not pre-assessed before allocation/vaccination. Mice (*n* = 6 per group) were vaccinated either subcutaneously with 40 μg of HA1–AP205dim or AP205dim (control) in 100 μL of PBS, or intranasally (i.n.) with 40 μg in a total volume of 50 μL (25 μL per nostril), without adjuvant. Mice were boosted with the same amount on day 28. Blood samples were taken weekly via the tail vein using Microtainer tubes (BD Biosciences, Franklin Lakes, NJ, USA). On day 49, both serum and bronchoalveolar lavage (BAL) samples were collected. Three weeks after the last vaccination, mice were anesthetised with isoflurane and intranasally challenged with mouse-adapted PR8 influenza virus (provided by Prof. Dr. Martin Kopf). Mice were monitored in a non-blinded manner for body weight, clinical symptoms, and survival rates for 14 days post challenge.

### 2.7. Enzyme-Linked Immunosorbent Assay (ELISA)

To evaluate antibody binding to the HA domain, 96-well ELISA plates (Corning) were coated with 1 µg/mL of H1N1 HA1 (A/Puerto Rico/8/1934) (ThermoFisher, Waltham, MA, USA, Cat. A42599) in PBS, 50 μL/well, and incubated at 4 °C overnight. Plates were rinsed five times with PBS and blocked with PBS/0.15% Casein (Sigma Aldrich, Burlington, MA, USA, C7078-500G) for 2 h at room temperature. Serum samples from immunised mice were serially diluted (1:3) in PBS/0.15% casein and incubated for 1 h at room temperature. Plates were washed five times with PBS+Tween 0.01%.

For detection, 50 μL of HRP-conjugated goat anti-mouse IgG antibody (Jackson ImmunoResearch, West Grove, PA, USA) or IgA (ICN 55549) at 1:1000 was added. Prior to IgA detection, serum IgG was depleted using Protein G beads (Invitrogen) as per the manufacturer’s instructions, to avoid interference via IgG detection. To analyse IgG subclasses, the following HRP-conjugated antibodies were used (all at 1:2000): rat anti-mouse IgG1 (BD Pharmingen, San Diego, CA, USA, Cat. 559626), biotinylated mouse anti-mouse IgG2a (Clone R19-15, BD Biosciences, Cat No 553391, USA), goat anti-mouse IgG2b (Invitrogen, Ref. M32407), and goat anti-mouse IgG3 (Southern BioTech, Birmingham, AL, USA, Cat No 1101-05). TMB (3,30,5,50-tetramethylbenzidine) substrate was used for colour development; reactions were stopped with 1 M of sulfuric acid and read at 450 nm immediately.

### 2.8. Avidity (ELISA)

To assess antibody avidity, two parallel ELISAs were performed as described above. After 1 h of the mice serum incubation step at RT, one plate was washed with PBS/Tween 0.01% and the other with 7 M of urea and incubated for 5 min to disrupt weakly bound antibodies. Plates were washed three times and processed as above. Then, 50 μL of HRP-conjugated goat anti-mouse IgG antibody (Jackson ImmunoResearch, West Grove, PA, USA) at a 1:1000 dilution was used as the detection antibody. TMB substrate and sulfuric acid were used to develop the plates as described above, and absorbance was measured at 450 nm. Avidity was calculated based on the OD values.

### 2.9. Murine Bronchoalveolar Lavage (BAL)

Bronchoalveolar lavage (BAL) was performed on day 49, as previously described by Sun et al. [30]. Briefly, mice were sacrificed via a lethal dose of sodium pentobarbital solution. Then, the trachea was surgically exposed and cannulated with a 22G catheter. Lungs were lavaged with 1 mL of PBS and 100 mM EDTA. Recovered BAL fluid was then centrifuged at 800× *g* for 10 min at 4 °C. Supernatants were transferred to fresh tubes and frozen at −20 °C [30].

### 2.10. Neutralisation

MDCK-II cells (provided by Georg Herrler, University of Veterinary Medicine, Hannover, Germany) were seeded onto 96-well plates and grown overnight before infection. Sera samples were ten-fold serially diluted in serum-free growth media (MEM, Life Technologies, Zug, Switzerland, Cat No 11095080) and incubated with 200 pfu of virus for 1 h at 37 °C. The mixture was then transferred onto the plate with the seeded cells in MEM, with 1% Trypsin (Sigma, Cat No T6763-250MG), 2% FBS (Pan Biotech, Aidenbach, Germany), and 1% (*v*/*v*) penicillin/streptomycin. Trypsin was added to facilitate the cleavage of the hemagglutinin (HA) protein, which is essential for retaining infectious influenza virus capable of entering new host cells. After overnight incubation, cells were washed twice with PBS and fixed in 3.6% formalin for 30 min at RT, quenched with 0.1 M of glycine, and permeabilised with 0.25% (*v*/*v*) Triton X-100 in PBS. Infected cells were stained using HB-65 anti-viral nucleoprotein (1:50 in PBS), followed by goat anti-mouse IgG conjugated to Alexa Fluor 488 (ThermoFisher, 1:500 in PBS). Fluorescence was visualised using an inverse fluorescence microscope (Zeiss, Germany). Neutralising antibodies (ND_50_) were calculated with the Spearman–Kärber formula [31].

### 2.11. Bio-Layer Interferometry (BLI) Assay

Antibody competition assays were performed using an Octet RED96e system (Fortébio). Anti-Penta-HIS biosensors (HIS1K, Lot 2006292) were pre-hydrated in BLI buffer (PBS, 0.1% BSA, and 0.02% Tween 20) for 10 min. Sensors were loaded with 10 μg/mL His-tagged H1N1 HA (A/Puerto Rico/8/1934(H1N1)) and loaded with mouse sera diluted 1:20. Regeneration was performed using 0.1 M of glycine (pH 1.5) followed by neutralisation with BLI buffer. Negative values were set to zero. K_dis_ values from monoclonal antibody control were below the detection limit (<10^−7^) of the instrument and were therefore set at 10^−7^ for statistical analysis.

### 2.12. Statistical Analyses

Analyses were conducted using one-way ANOVA as well as Student’s *t*-tests. Survival analyses were compared using the Mantel–Cox log-rank test. A *p*-value of less than 0.05 was considered statistically significant. All statistical analyses were conducted using GraphPad Prism version 10.00 (GraphPad Software, San Diego, CA, USA). Statistical significance is indicated as follows: *p* < 0.05 (* *p* < 0.05, ** *p* < 0.01, *** *p* < 0.001, **** *p* < 0.0001), *p* > 0.05 (ns, no significant difference).

## 3. Results

### 3.1. HA1–AP205dim Vaccine Production

**Figure 1:**
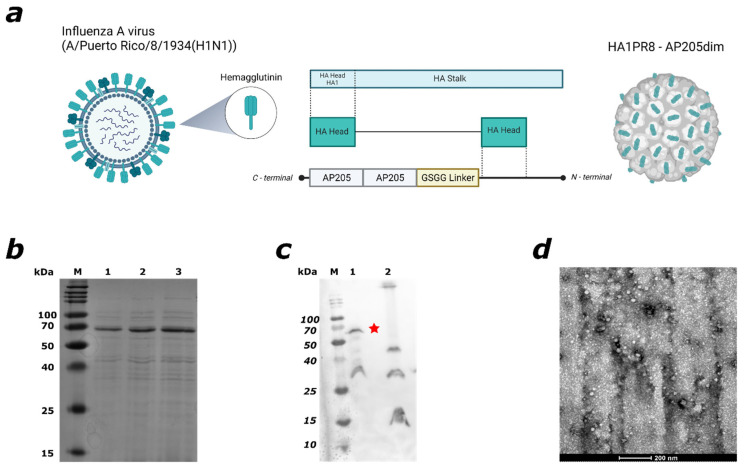
Design, production, and verification of the HA1–AP205dim fusion vaccine. (**a**) Schematic representations of the generation of the HA1–AP205dim vaccine. In a first step, the HA1 head domain of A/Puerto Rico/8/1934/H1N1 was genetically fused to the N-terminus of dimerised capsid protein. The capsid protein consists of a dimerised AP205dim coat protein. (**b**) SDS-PAGE analysis (12%) of the purified HA1–AP205dim fusion protein, showing a prominent band at the expected molecular weight. M, protein marker; lanes 1–3, purified HA1–AP205dim. (**c**) Western blot conducted with α-AP205 antibody, with AP205 as control VLP. M: protein marker; 1: HA1–AP205dim; and 2: AP205 control. The Western blot confirms the correct size of the HA1 to AP205dim fusion (Red Star). (**d**) Transmission electron microscopy (TEM) image of the purified HA1–AP205dim VLPs, scale bar is set to 200 nm. Figure (**a**) was created with Biorender.com (accessed on Q1–Q2 2025).

To develop a potent and scalable vaccine, we focused our design on the hemagglutinin (HA) surface protein, the principal target of neutralising antibodies. These antibodies (Abs), mainly directed against the HA head domain, block viral binding to host cells and protect against influenza infection [32]. In this study, the head domain (HA1) of the A/Puerto Rico/8/1934(H1N1) strain was genetically fused to a dimerized version of the AP205 coat protein (AP205dim) using flexible GSGG linker (Figure 1a). This design enables the entire HA1 domain to be displayed on the surface of virus-like particles (VLPs), taking advantage of the dense and repetitive presentation of epitopes characteristic of AP205-based scaffolds. The fusion protein was successfully expressed in *E. coli*, a cost-effective system suitable for large-scale vaccine production [23,24]. After expression, the protein was isolated from inclusion bodies and refolded using a urea gradient. SDS-PAGE analysis confirmed the presence of a protein band at the expected molecular weight (67 kDa) for HA1–AP205dim (Figure 1b). Western blotting with an anti-AP205 antibody validated the identity of the fusion construct (Figure 1c), showing a specific band corresponding to HA1–AP205dim. The protein was further purified and concentrated; its structural integrity was analysed using an electron microscope (Figure 1d). The results confirm that the HA1 sequence was successfully incorporated into the AP205dim scaffold.

### 3.2. HA1–AP205dim Vaccine Elicits a High Amount of Specific Antibodies Against the HA Protein in Sera

**Figure 2:**
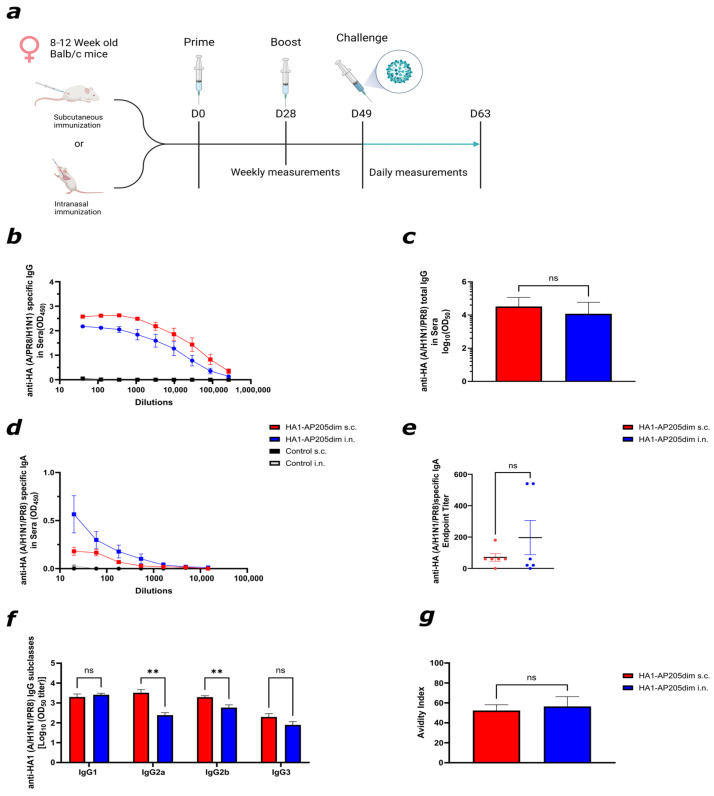
Subcutaneous and intranasal immunisation with the HA1–AP205dim vaccine induced strong specific antibodies in sera. (**a**) Schematic of the vaccination and sampling timeline. Mice were immunised on day 0 and boosted on day 28 with 40 μg of HA1–AP205dim either subcutaneously (s.c.) or intranasally (i.n.). Sera were collected weekly and analysed on day 49. After day 49, mice were challenged with a 2 × LD50 dosage of A/PR8/H1N1 mouse-adapted influenza virus and monitored for two weeks. (**b**,**c**) Serum IgG responses to recombinant HA protein (A/Puerto Rico/8/1934(H1N1)) measured via ELISA. Log-transformed OD50 values were calculated from dilution curves. (**d**,**e**) Specific serum IgA responses to HA, assessed via endpoint titre. (**f**) IgG subclasses in blood sera. HA1–A/PR8/H1N1-specific IgG-subclass titres on day 49 measured via ELISA Log_10_ (OD_450_) shown in either vaccinated s.c. or i.n. (**g**) Avidity index of HA-specific IgG in serum. Statistical analysis (mean ± SEM) using Student’s *t*-test. *p* ≤ 0.01 (**). One representative graph is shown. Vaccine and control groups *n* = 6. Figure (**a**) was created with Biorender.com (accessed on Q1–Q2 2025).

To evaluate the immunogenicity of the HA1–AP205dim vaccine, BALB/c mice were immunised either subcutaneously (s.c.) or intranasally (i.n.) with 40 μg of vaccine on day 0, followed by a homologous booster on day 28 (Figure 2a). Control groups received AP205dim particles without the HA1 insert via the same routes. Serum samples were collected weekly and humoral responses were analysed on day 49. Both vaccination routes elicited strong antibody responses against HA. Furthermore, mice immunised with HA1–AP205dim exhibited robust serum IgG reactivity against HA(OD_450_), with comparable total IgG levels (Log_10_(OD_450_)) following both subcutaneous (s.c.) and intranasal (i.n.) administration (Figure 2b,c). Similarly, significantly elevated levels of HA-specific serum IgA were observed in both groups (Figure 2d,e), indicating that intranasal immunisation can induce systemic humoral responses that are comparable to those induced via subcutaneous administration. However, not all immunised mice developed detectable IgA titres, suggesting some variability in response. Mice immunised with AP205dim VLP, without the genetically fused HA1, showed no induction of specific antibodies. For a more comprehensive assessment of the quality of the antibodies, the distribution of IgG subclasses was analysed. Subcutaneous vaccination induced significantly higher levels of IgG2a and IgG2b compared to intranasal delivery, while levels of IgG1 and IgG3 remained comparable between the groups (Figure 2f). This profile suggests a more pronounced Th1-skewed response following s.c. administration, consistent with enhanced class switching [27]. The avidity of the IgG response was then assessed using a urea-based ELISA. High-avidity antibodies were detected in both vaccination groups, with no significant difference in avidity index between the intranasal (i.n.) and subcutaneous (s.c.) delivery routes (Figure 2g). These findings indicate that both administration methods elicit functionally mature antibody responses, implying that affinity maturation enhances antibody avidity and functional quality [33].

### 3.3. HA1–AP205dim Induces Local IgG and IgA Responses in the Bronchoalveolar Lavage Fluid

**Figure 3:**
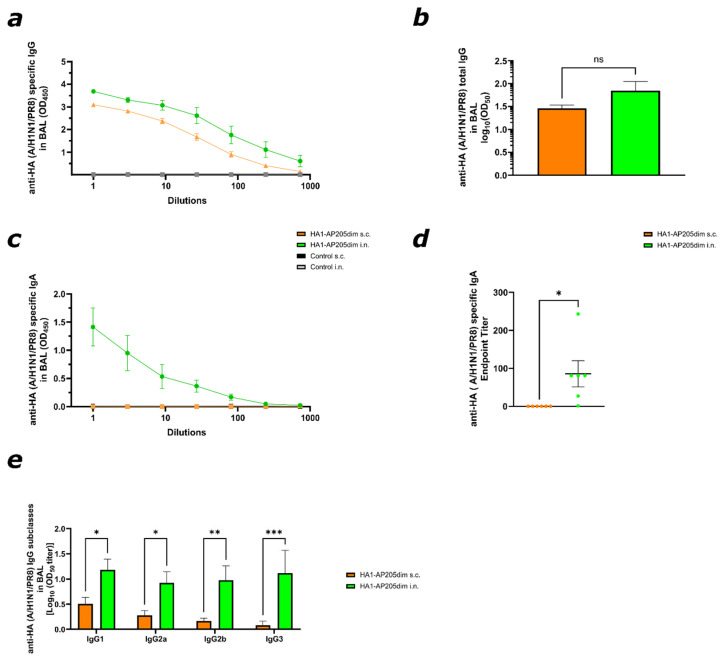
Subcutaneous and intranasal immunisation with HA1–AP205dim induces strong specific antibody in the bronchoalveolar fluid (BAL). (**a**,**b**) HA–A/PR8/H1N1-specific IgG in BAL on day 49 measured via ELISA in either vaccinated s.c. or i.n. AP205dim was used as a control vaccination, both s.c. and i.n. (**c**,**d**) HA-specific IgA endpoint titre in BAL. IgA was detected only in i.n.-immunised mice. (**e**) IgG subclasses in BAL. HA1–A/PR8/H1N1-specific IgG-subclass titres on day 49 measured via ELISA Log_10_ (OD_450_) shown in either vaccinated s.c. or i.n. Statistical analysis (mean ± SEM) using Student’s *t*-test. *p* ≤ 0.05 (*), *p* ≤ 0.01 (**) and *p* ≤ 0.001 (***). Control group *n* = 3 (s.c., i.n.), vaccine group *n* = 6 (s.c., i.n.). For (**e**) vaccine group *n* = 5 (i.n.) and *n* = 6 (s.c.) (BAL).

To evaluate local mucosal immunity, bronchoalveolar lavage (BAL) fluid was collected from vaccinated mice on day 49. HA-specific antibody responses in BAL were assessed via ELISA. Both i.n. and the subcutaneous s.c. delivery of HA1–AP205dim resulted in the induction of HA-specific IgG in bronchoalveolar lavage (BAL) fluid. Although IgG levels were slightly higher in mice immunised via the intranasal route, this difference did not reach statistical significance (see Figure 3a,b). In contrast, HA-specific IgA was only observed in mice immunised via the intranasal route, whereas subcutaneously vaccinated mice showed no detectable IgA response (see Figure 3c,d). No HA-specific IgG or IgA was detected in BAL from control mice immunised with AP205dim without the HA1 insert.

IgG subclass analysis revealed a balanced induction in the bronchoalveolar lavage (BAL) of intranasal (i.n.) immunised mice, whereas subcutaneous (s.c.) immunised animals showed significantly lower levels across all subclasses (Figure 3e). This profile contrasts with the systemic response, indicating a distinct local antibody distribution following mucosal vaccination. Altogether, these findings demonstrate that while both immunisation routes induce local IgG in the respiratory tract, only intranasal vaccination elicits detectable mucosal IgA and robust IgG subclass responses in BAL. This finding aligns with previous studies demonstrating that intranasal VLP-based vaccines can effectively induce local mucosal IgA and IgG responses [34].

### 3.4. HA1–AP205dim Induces HA-Binding and Virus-Neutralising Antibodies

To evaluate the binding quality and functional activity of vaccine-induced antibodies, serum samples collected on day 49 were analysed using bio-layer interferometry (BLI) and virus neutralisation tests (VNT).

**Figure 4:**
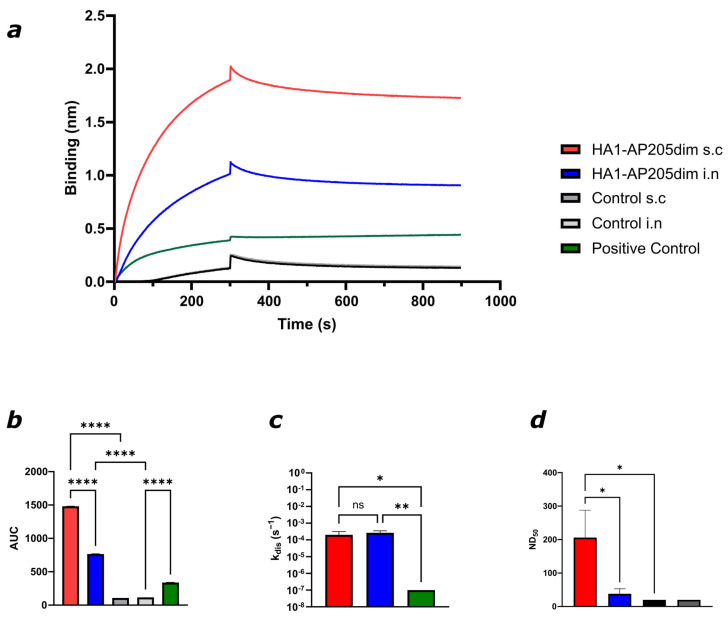
HA1–AP205dim-induced antibodies can bind efficiently to the HA protein and can neutralise (H1N1/A/PR8/1934) virus in vitro. (**a**,**b**) Average BLI sensorgrams generated from six individual replicates showing binding of sera from mice immunised intranasally (i.n.) or subcutaneously (s.c.) with HA1–AP205dim to recombinant HA protein (A/Puerto Rico/8/1934(H1N1)). A monoclonal antibody against the HA1 domain was used as a positive control with four individual replicates. Sera from AP205dim-immunised mice served as negative controls (*n* = 6). (**c**) Comparison of dissociation rate constants between sera from s.c.- and i.n.-immunised mice and a monoclonal antibody. The decrease in signal was recorded during the dissociation phase in the buffer. (**d**) Virus neutralisation test (VNT) against A/Puerto Rico/8/1934(H1N1) virus. Neutralisation titres are reported as the serum dilution that achieves 50% neutralisation (ND_50_). One representative graph is shown. Statistical analysis (mean ± SEM) using one-way ANOVA. *p* ≤ 0.05 (*), *p* ≤ 0.01 (**), and *p* ≤ 0.0001 (****). Control group *n* = 6 (s.c., i.n., sera), vaccine group *n* = 6 (s.c., i.n., sera), and *n* = 4 (positive control, mAb).

BLI was performed using recombinant HA protein from A/Puerto Rico/8/1934(H1N1) immobilised on biosensors. Sera from both intranasally (i.n.) and subcutaneously (s.c.) immunised mice showed strong binding to the HA-coated biosensor. In contrast, control sera from mice immunised with AP205dim particles devoid of HA1 exhibited no distinct binding. A monoclonal antibody specific for the HA1 domain was included as a positive control and demonstrated robust binding to the HA-coated sensors. A comparison of the binding kinetics between the two immunisation routes was conducted through the analysis of the area under the curve (AUC) of the sensorgrams (see Figure 4a,b). The results showed that sera from s.c.-immunised mice displayed higher binding than those from i.n.-immunised mice, suggesting a stronger and more sustained antibody response to the HA antigen after subcutaneous immunisation.

To assess the stability of antigen–antibody interactions elicited by different immunisation routes, we compared the dissociation rate constants to a reference monoclonal antibody. The dissociation rate reflects the stability of the formed immune complexes, with lower s^−1^ values indicating more stable interactions (Figure 4c). Both s.c.- and i.n.-immunised mouse sera exhibited comparable dissociation rates in the range of ~10^−4^, with no significant difference between the two groups. In contrast, the monoclonal antibody showed a significantly lower dissociation rate (<10^−7^), indicating significantly more stable binding. This observation may be attributable to the presence of polyclonal antibodies in the sera, which exhibit a reduced binding capacity relative to monoclonal antibodies.

The functional neutralisation of sera was evaluated using a virus neutralisation test (VNT) against the mouse-adapted A/Puerto Rico/8/1934(H1N1) virus in MDCK cells. Neutralising activity was expressed as the 50% neutralisation dose (ND_50_), calculated from serial dilutions. Both immunisation routes induced neutralising antibodies, with significantly higher titres observed in the s.c. group (Figure 4d). No neutralisation was observed in control sera. These results confirm that HA1–AP205dim induces high-affinity, functional antibodies capable of binding HA antigen and neutralising influenza virus, independent of the route of administration.

### 3.5. HA1–AP205dim Vaccination Protects Mice Against Lethal Influenza Challenge

**Figure 5:**
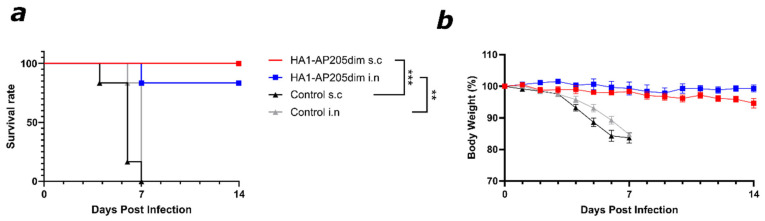
Immunised mice with HA1–AP205dim are protected against A/PR8/H1N1 live virus challenge. (**a**) Survival rate of intranasally challenged mice. Mice were challenged with 2 × LD_50_ of live virus for 14 days. (**b**) Body weight difference from day 0 until day 14 and survival rate. We showed that s.c. and i.n. immunizations of mice with HA1–AP205dim vaccines prevent body weight loss and confer protection against a lethal challenge with A/PR8/H1N1 virus. Body weight change (%) following viral challenge over 14 days, last point carried forward. One representative graph is shown. Data represent mean ± SEM. Survival was analysed via the Mantel–Cox log-rank test, *p* values are denoted as follows: *p* ≤ 0.01 (**), *p* ≤ 0.001 (***). Control group *n* = 6 (s.c., i.n.); vaccine group *n* = 6 (s.c., i.n.).

To assess the protective efficacy of the HA1–AP205dim vaccine in vivo, vaccinated and control mice were challenged intranasally with 2×LD50 of the mouse-adapted A/Puerto Rico/8/1934(H1N1) virus, three weeks after the final immunisation. Mice were then monitored daily over 14 days for changes in body weight, clinical signs of disease, and survival. Remarkably, almost all mice immunised with HA1–AP205dim, either intranasally (i.n.) or subcutaneously (s.c.), were largely protected against mortality, with only one vaccinated mouse succumbing to infection (Figure 5a). In contrast, all control animals immunised with AP205dim lacking the HA1 insert died to infection by day 8 post challenge. Furthermore, the vaccinated mice exhibited minimal or no weight loss and maintained body weights close to baseline throughout the monitoring period (Figure 5b). Control animals exhibited rapid and significant weight loss, consistent with progressive viral disease. These results demonstrate that HA1–AP205dim protects against lethal influenza challenge in mice, regardless of the route of administration.

## 4. Discussion

In this study, the immunogenicity and protective efficacy of a virus-like particle (VLP) vaccine displaying the HA1 domain of A/Puerto Rico/8/1934(H1N1) on the AP205dimer platform were evaluated. The present study demonstrates that HA1–AP205dim induces potent systemic and mucosal antibody responses, conferring protection against a lethal influenza challenge in a murine model.

The subcutaneous (s.c.) and intranasal (i.n.) immunisation routes were found to be equally efficacious in eliciting specific IgG and IgA levels in sera. Intranasal vaccination has been demonstrated to elicit mucosal IgA responses in bronchoalveolar lavage (BAL). This finding underscores the capacity of this administration modality to elicit local immunity at the site of viral entry, a capacity which has already been shown with other VLP-based vaccines [34,35]. The potential of the intranasal route to elicit both systemic and mucosal responses makes it a valuable tool in the management of respiratory pathogens such as influenza [36,37].

Further characterisation of the antibody response revealed high-avidity IgG antibodies capable of binding HA and neutralising virus in vitro. It is essential to note that both immunisation routes resulted in the production of functionally competent antibodies, as evidenced by bio-layer interferometry (BLI) and virus neutralisation tests (VNT). The dissociation rates of the two administration routes showed no significant difference. However, the intranasal vaccination resulted in lower overall binding and neutralisation titres than the subcutaneous route. These findings suggest that both routes are protective; subcutaneous immunisation may elicit a quantitatively stronger antibody response in sera, perhaps due to more efficient systemic priming and enhanced antigen presentation [33]. Indeed, previous studies have shown that although intranasal immunisation efficiently induces mucosal IgA responses, it typically results in lower systemic IgG titres than parenteral routes [38]. Nevertheless, this does not necessarily compromise protective efficacy, as both our findings and earlier reports demonstrate that mucosal immunisation can confer equivalent protection, likely through local immune responses and secretory IgA at the site of viral entry [39].

The vaccine’s protective efficacy was demonstrated in vivo, with vaccinated mice experiencing minimal weight loss and high survival rates after a lethal viral challenge. These findings support the use of AP205-based VLPs as a flexible platform for scalable, cost-effective vaccine production in bacterial systems. Their repetitive antigen display and encapsulated prokaryotic RNA, acting as a natural TLR7/8 ligand, enable strong immune responses without added adjuvants [24]. The AP205dim platform may also offer potential for addressing zoonotic influenza threats through targeted immunisation strategies in high-risk human populations and animal reservoirs. In recent years, several VLP-based influenza vaccine candidates have been developed using mammalian, baculovirus–insect, plant, or bacterial expression systems [24]. While most have showed non-inferior immunogenicity compared to the existing inactivated/recombinant vaccines, but broad superiority, particularly in terms of high efficacy in the elderly, the durability of protection and production costs remains to be established.

In this context, the AP205dim platforms offers distinct advantages: low-cost manufacturing, intrinsic adjuvanticity, balanced systemic and mucosal immunity, and the ability to incorporate the full-length HA1 domain. The latter preserves conformational epitopes that may be lost in truncated constructs, enhancing antibody breadth and quality [20]. This design promotes high-affinity IgG and mucosal IgA responses, the latter being critical for blocking respiratory pathogens such as influenza at the site of entry [20]. This adaptability, combined with its production efficiency and scalability, supports its use for seasonal vaccination, rapid response to emerging influenza strains, and applications in both human and veterinary medicine. Future research should explore the breadth of protection conferred by HA1–AP205dim, including cross-reactivity to antigenically drifted strains and performance in multivalent or combination formulations.

A limitation of the study is that it was tested with only one strain of the influenza virus. We are currently developing an H5 vaccine based on the AP205dim platform, along with other VLP-based influenza vaccine candidates. Furthermore, although the AP205dim platform enables bacterial production of complex vaccine antigens, further optimisation of expression, purification, and refolding processes is needed to improve yields and streamline production for large-scale applications. In addition, all experiments to date have been conducted in a mouse model, which may not fully reflect vaccine performance in humans and other relevant species such as ferrets. Further experiments are planned to expand the antigenic breadth of the platform, including the incorporation of additional targets such as the matrix 2 ectodomain (M2e), neuraminidase (NA), and conserved HA stalk domains.

In summary, HA1–AP205dim is proving to be a highly promising and strategically versatile vaccine candidate. Its ability to induce robust systemic and mucosal immunity, as demonstrated in both immunogenicity and protection studies, represents a significant advancement in influenza vaccine development. The inherent modularity of the AP205dim flexible antigen exchange supports timely adaptation to novel or emerging strains. Coupled with its adjuvant-free formulation and low-cost bacterial production, this platform offers a practical and scalable solution. These advantages are particularly relevant in the context of coordinated One Health strategies, where integrated efforts across human and veterinary medicine are essential to reduce transmission risks and respond effectively to evolving influenza threats [8]. Low-cost production is particularly important in the veterinary sector, in particular in poultry. Taken together, these findings support the continued development of HA1–AP205dim as a promising candidate for influenza preparedness and control, warranting further evaluation in preclinical and clinical settings.

## Data Availability

The original contributions presented in this study are included in the article. Further inquiries can be directed to the corresponding author(s).
